# The Treatment of Acute Summer Diarrhœa in Infants

**Published:** 1905-08-19

**Authors:** 


### HOSPITAL CLINICS.

### THE TREATMENT OF ACUTE SUMMER DIARRHŒA IN INFANTS.

ACUTE infantile summer diarrhœa, in the form of widespread epidemics which add seriously to our excessive infant mortality, is an only too familiar experience. Diarrhœa and vomiting of sudden onset and rapidly leading to profound collapse are the leading features of the disease. Another distinguishing fact is the rapidity with which the tissues are rapidly drained of their fluid, so that in the course of a few hours a previously healthy infant is found with wrinkled skin, depressed fontanelle, sunken eyes, cold breath, and an almost inaudible cry. The course of the temperature is of importance in relation both to diagnosis and prognosis. Raised at the outset, it usually falls to subnormal in the condition of collapse. Then, if recovery from the collapse manifests itself, there is a temporary rise above the normal level. Persistence of this rise, or again, a continued high temperature without collapse, are facts of bad prognostic omen. On the other hand, though other indications may appear unfavourable there is always a chance of recovery when the temperature is normal. An interesting observation in some cases is the persistence of pyrexia after diarrhœa and vomiting have ceased, a significant indication of the toxæmic nature of the disease.

For treatment in the early stages, the wise plan is to give a purgative and temporarily to withhold all milk. In the milder varieties castor oil acts admirably; but calomel is more suitable in acute cases. It is less likely to be vomited, has some value as an intestinal antiseptic, and may act indirectly as a diuretic—the last-mentioned being a consideration of importance when renal activity is defective. The purgative at the outset is of undoubted value in removing deleterious substances from the intestine, but when vomiting and diarrhœa have persisted for some time, and milk has either not been administered or not retained, purgation is a doubtful, possibly a harmful, process. For the vomiting, which is usually the earliest symptom, no measure is so successful as washing out the stomach with warm water or a weak solution of sodium bicarbonate introduced through a soft tube passed through the nose. Afterwards, one-tenth to one-eighth of cocaine in a teaspoonful of iced water may be given. In slighter cases cocaine given in this fashion may render lavage unnecessary. Other useful therapeutic measures are the application of a mustard poultice to the epigastrium, frequent teaspoonfuls of hot water, and bismuth carbonate and sodium bicarbonate (not less than five grains of each, even to the youngest infant), with quarter minim doses of dilute hydrocyanic acid. If, after a period of abstention from food, the vomiting recurs when food is resumed, the latter should be given entirely cold or even iced. The numerous drugs recommended to check the diarrhœa are none of them absolutely to be trusted, and so far as the direct astringents are concerned it may be granted that they are of little or no value in the early stages of the disorder, though they have their use in the more chronic diarrhœa which often persists after the acute phase. To correct the fœtor of the motions a mixture containing a minim each of glycerine of carbolic acid, and tincture of iodine is usually successful in the course of one to two days, and when this end has been attained, a half grain each of grey powder and Dover's powder given every four hours, alternately with the bismuth mixture as above, commonly checks the diarrhœa. With persisting diarrhœa and no tendency to vomiting zinc oxide may be ordered as well as the mercury with Dover's powder. With continued pyrexia treatment is rarely successful, but calomel and Dover's powder, one-tenth to one-eighth grain of each, is on the whole the most promising medicinal combination. The wet pack is also of value and in all cases where diarrhœa persists a daily irrigation of the large bowel with saline solution should be practised for three or four days.

To meet the condition of collapse the hypodermic injection of strychnine is indicated. A minim may be given to an infant of nine or ten months and repeated as necessary. In the same circumstances a mustard bath is of great value. Besides its general stimulating action it helps to restore the function of the skin, and sweating may be taken as a decidedly favourable sign. After the bath the lower bowel should be washed out with a pint of normal saline solution at 105°. This should be introduced by an irrigator and will in part be retained and act as a restorative. When part is rejected a second injection of about four ounces should be ordered and to this, if necessary, some alcoholic stimulant may be ordered. These smaller injections may be repeated every three or four hours. In every stage of the disease the question of feeding is of the first importance. For the first twelve hours or so all food should be withheld and only small doses of hot water permitted, though, if the condition of the patient is urgent, small quantities of white wine whey can be tried. This is often retained when all other substances are rejected, but it must be remembered that the proportion of nutrient material it contains is small and that of alcohol relatively large for an infant. During the period in which milk is suspended, in the earlier phases of the illness, veal or mutton broth, albumin-water, and raw meat-juice are the best temporary substitutes, though they should not be employed for longer than 24 or, at the outside, 48 hours. Of these, raw meat-juice, more especially a fresh, home-made preparation, has the highest nutritive value, but if used in quantity for more than a few days it is apt to give rise to foul, tarry motions. After its use, therefore, for one or two days it must be replaced by some substitute such as Mellin's food with whey, or a good brand of unsweetened condensed milk may be tried. To commence, one part of the milk may be diluted with four parts each of barley-water and lime-water and the mixture slightly sweetened. Then the proportion of milk may be gradually increased, and perhaps in the course of a few days boiled or sterilised cow's milk may be substituted for the condensed variety. If this proves a failure a return must be made to the condensed milk, and though this is not a perfect substitute for cow's milk many infants thrive on it fairly well even when its use is continued for long periods. Indeed, in many cases of diarrhœa it is a useful policy to replace the ordinary diet of cow's milk for a few days by a good brand of unsweetened condensed milk.

The prevention of the acute summer diarrhœa of infants demands recognition of the more extreme bacteriological contamination that milk undergoes in hot weather. Hence the occurrence of the disease mainly in hand-fed children. It follows, then, that in hot weather all cow's milk to be used in feeding infants should be boiled or, still better, sterilised. A contributing influence is the enervating tendency exercised by high temperatures on the digestive apparatus. Hence during the hottest months it is well to lengthen the interval between the feedings, this applying also to breast-fed infants, and also to lessen both the quantity and strength of the cow's milk. Any discomfort attending this regulation may be met by occasional small doses of plain water. Lastly, any diarrhœa, however slight, which occurs in summer should receive prompt attention. A dose of castor oil and a few hours' suspension of food will often prove a safeguard against more severe developments.

[1] Dr. J. A. Coutts: Lancet, July 29, 1905.

